# Supplementary figures and images for: Mapping the Anti-Cancer Activity of α-Connexin Carboxyl-Terminal (aCT1) Peptide in Resistant HER2+ Breast Cancer
Source: Cancers (Basel). 2024 Jan 19;16(2):423. doi: 10.3390/cancers16020423 (PMC10814893; doi:10.3390/cancers16020423)

# Figure 2

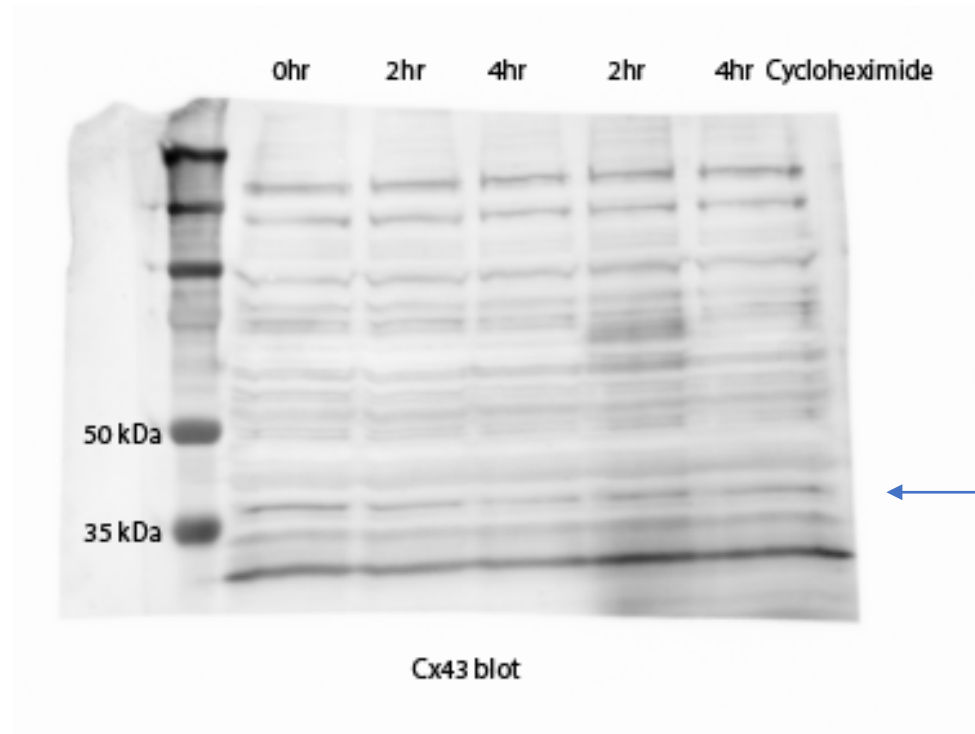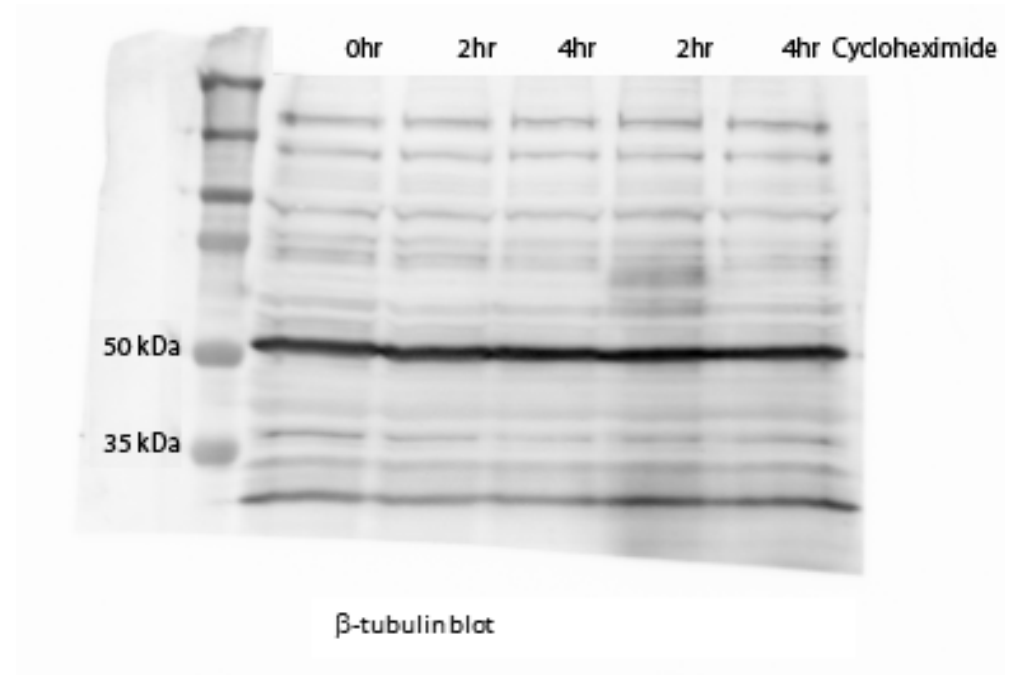

# Figure 3

ZO1 blot

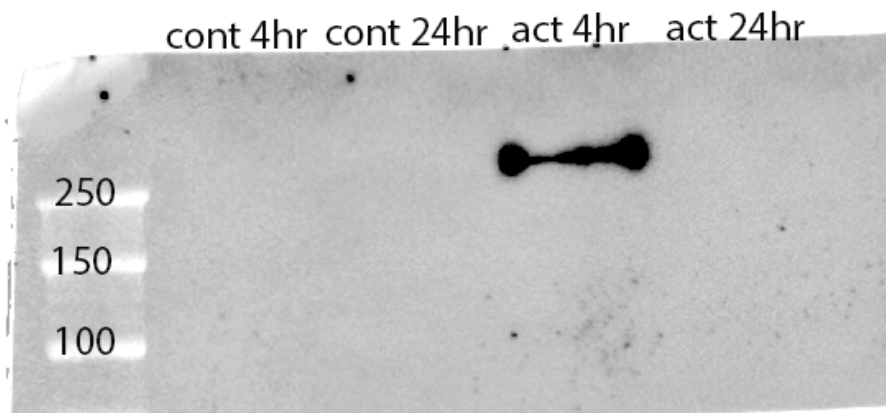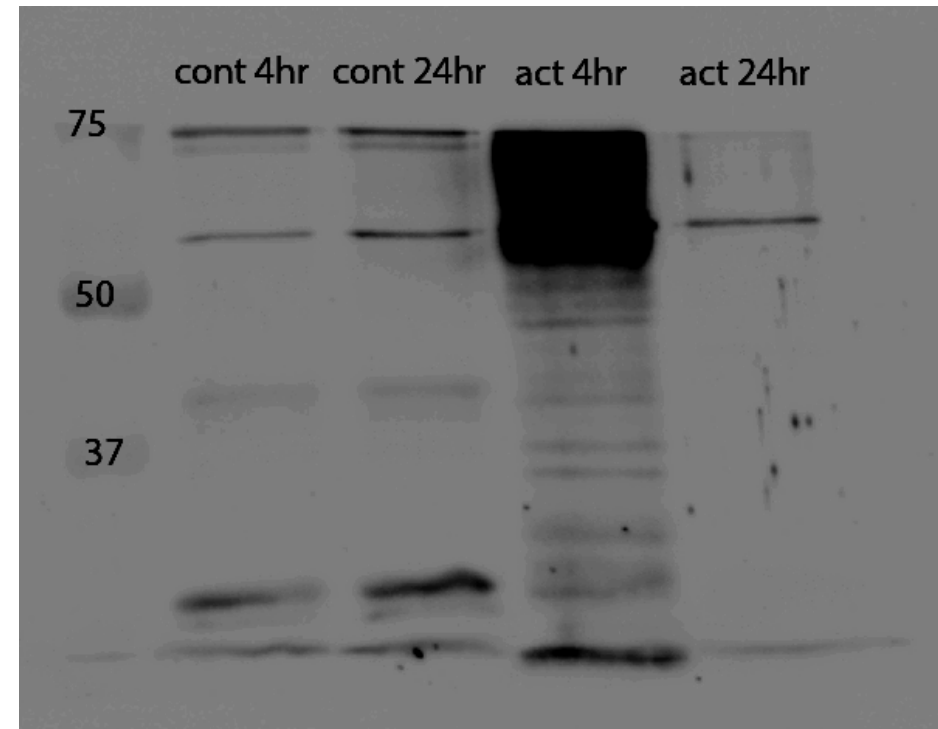

Supplement: Supplementary file 1 [file cancers-16-00423-s001.zip › cancers-2809553-original-images.pdf]
